# Growth hormone-releasing hormone-secreting pulmonary neuroendocrine tumor associated with pituitary hyperplasia and somatotropinoma

**DOI:** 10.20945/2359-3997000000395

**Published:** 2021-09-29

**Authors:** Elisa B. Lamback, Daniel G. Henriques, Mari C. Vazquez-Borrego, Carlos H. de Azeredo Lima, Leandro Kasuki, Raul M. Luque, Leila Chimelli, Mônica R. Gadelha

**Affiliations:** 1 Hospital Universitário Clementino Fraga Filho Faculdade de Medicina Divisão de Endocrinologia Rio de Janeiro RJ Brasil Centro de Pesquisa em Neuroendocrinologia, Divisão de Endocrinologia, Faculdade de Medicina, Hospital Universitário Clementino Fraga Filho, Rio de Janeiro, RJ, Brasil; 2 Instituto Estadual do Cérebro Paulo Niemeyer Laboratório de Neuropatologia e Genética Molecular Rio de Janeiro RJ Brasil Laboratório de Neuropatologia e Genética Molecular, Instituto Estadual do Cérebro Paulo Niemeyer, Secretaria Estadual de Saúde, Rio de Janeiro, RJ, Brasil; 3 Instituto Estadual do Cérebro Paulo Niemeyer Unidade de Neuroendocrinologia Rio de Janeiro RJ Brasil Unidade de Neuroendocrinologia, Instituto Estadual do Cérebro Paulo Niemeyer, Secretaria Estadual de Saúde, Rio de Janeiro, RJ, Brasil; 4 Instituto Maimónides de Pesquisas Biomédicas de Córdoba Córdoba Espanha Instituto Maimónides de Pesquisas Biomédicas de Córdoba (IMIBIC), Córdoba, Espanha; 5 Universidade de Córdoba Departamento de Biologia Celular, Fisiologia e Imunologia Córdoba Espanha Departamento de Biologia Celular, Fisiologia e Imunologia, Universidade de Córdoba, Córdoba, Espanha; 6 Hospital Universitário Reina Sofia Córdoba Espanha Hospital Universitário Reina Sofia (HURS), Córdoba, Espanha; 7 CIBER Fisiopatologia da Obesidade e Nutrição Córdoba Espanha CIBER Fisiopatologia da Obesidade e Nutrição (CIBERobn), Córdoba, Espanha; 8 Hospital Federal de Bonsucesso Divisão de Endocrinologia Rio de Janeiro RJ Brasil Divisão de Endocrinologia, Hospital Federal de Bonsucesso, Rio de Janeiro, RJ, Brasil

## Abstract

Acromegaly caused by ectopic growth hormone-releasing hormone (GHRH)-secreting tumor is exceedingly rare. We report a case of acromegaly secondary to GHRH secretion by an incidentally diagnosed pulmonary neuroendocrine tumor (NET) and review 47 similar cases in literature. A 22-year-old male patient presented with symptoms of pituitary apoplexy. Magnetic resonance imaging (MRI) showed apoplexy of a pituitary adenoma. Routinely prior to surgery, a chest radiography was performed which revealed a mass in the left lung. During investigation, the patient was diagnosed with metastatic GHRH-secreting pulmonary NET. In retrospect, it was noted that the patient had pituitary hyperplasia 20 months prior to the MRI which showed the presence of a pituitary adenoma. The histological findings confirmed somatotroph hyperplasia adjacent to somatotropinoma. This case suggests that GHRH secretion can be associated with pituitary hyperplasia, which may be followed by pituitary adenoma formation.

## INTRODUCTION

Acromegaly is a chronic disease due to excess growth hormone (GH) and insulin-like growth factor type I (IGF-I) ( 1 ). GH-secreting pituitary adenomas (somatotropinomas) are responsible for the vast majority of cases, while ectopic GH-releasing hormone (GHRH) secretion is exceedingly rare accounting for less than 1% of cases ( 2 ). In the context of ectopic secretion, acromegaly is caused by pulmonary neuroendocrine tumors (NET) in majority of cases, followed by gastro-enteropancreatic NET, and rarely by pheochromocytomas or paragangliomas ( 3 ). Pulmonary NET are uncommon, having an incidence of 0.2 to 2 per 100,000, and are classified as typical carcinoid, atypical carcinoid, large cell neuroendocrine carcinoma and small cell neuroendocrine carcinoma based on 2015 World Health Organization (WHO) classification ( 3 , 4 ). About 70 cases of ectopic secreting GHRH tumors have been described to date with pulmonary NETs representing the majority of cases ( 5 ).

The clinical features of ectopic acromegaly are indistinguishable from those resulting from somatotropinoma ( 5 ). However, some findings are suggestive of ectopic secretion such as pituitary hyperplasia which is related to prolonged GHRH hypersecretion, local tumoral symptoms (from airway obstruction in the case of pulmonary NETs), cosecretion of humoral factors synthetized by well-differentiated NETs causing carcinoid syndrome ( 6 , 7 ). Excessive circulating serotonin levels leads to carcinoid heart disease, diarrhea and abdominal pain ( 7 ).

We report a case of a male patient who was diagnosed with acromegaly due to GHRH-secreting pulmonary NET and somatotropinoma, who presented with carcinoid syndrome, and review cases of GHRH-secreting pulmonary NETs published in literature.

This study was conducted in accordance with the ethical standards of the Helsinki Declaration, with the approval of the *Instituto Estadual do Cérebro Paulo Niemeyer* Ethics Committees involved in the study (CAAE 39632820.8.0000.8110). Patient's written consent was obtained for publication.

## CLINICAL SUMMARY

### Clinical presentation

**Figure 1:**
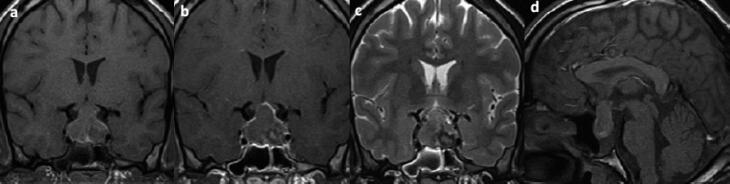
MRI at 22 years of age in coronal T1W (a), post-contrast T1W (b), coronal T2W (c) and sagittal T1W (d) views showing a 3.3 × 2.4 × 1.4 cm sellar lesion, with suprasellar extension and optic chiasm compression, heterogeneous, predominantly isointense in T1W and T2W, with peripheral contrast enhancement, exhibiting an internal small area with hypointense rim in T2W, suggestive of pituitary adenoma with hemorrhagic degeneration in different stages of evolution.

A 22-year-old male patient presented with sudden and severe headache after dental extraction. Brain computed tomography (CT) showed an isodense pituitary lesion, with no contrast enhancement. No visual disturbance was present. Sellar region magnetic resonance imaging (MRI) performed nine days after the headache revealed a 3.3 × 2.4 × 1.4 cm lesion suggestive of pituitary apoplexy (PA) ( Figure 1 ). The patient exhibited mild acromegaly features with mandibular prognathism, prominent forehead and tall stature that was familial.

Of note, the patient had a brain MRI performed 20 months before due to mental confusion and dizziness that showed slight enlargement of pituitary gland of 9.31 mm (normal range adjusted for sex and age: 5.63 + SDS 1.00) ( 8 ). The patient also complained of occasional abdominal cramps and diarrhea that started one year before and had an unexplained weight loss of 10 kg during the last two months. The patient had no family history of endocrine tumor.

On biochemical assessment, the patient had increased IGF-I levels of 823 ng/mL (normal range 99-289) and basal GH levels of 2.05 ng/mL, panhypopituitarism [free thyroxine 0.60 ng/dL (0.70-1.90), total testosterone < 10 ng/dL (220-819), cortisol 0.59 mcg/dL]. Diluted prolactin was 2.20 ng/mL (2.00-15.20). The patient was diagnosed with acromegaly and submitted to transsphenoidal surgery with complete removal of the pituitary lesion. The post-operative course was uneventful.

**Figure 2:**
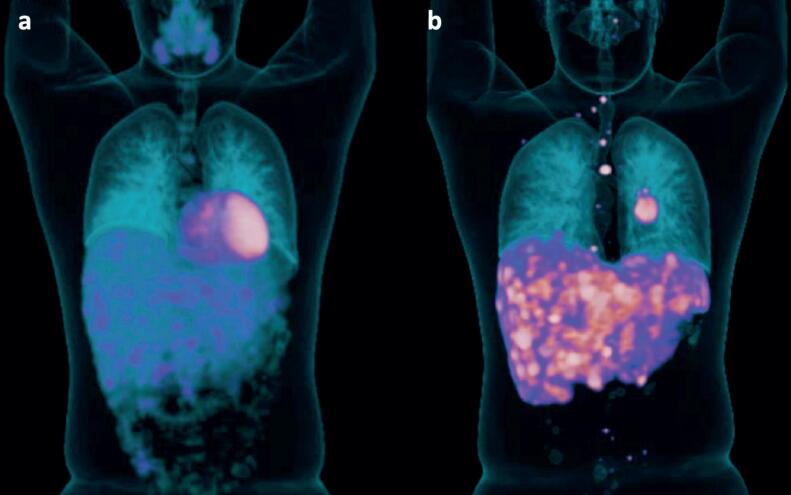
^18^F-FDG PET CT in coronal view (a) and ^68^Ga-DOTATOC PET CT in coronal view (b). ^18^F-FDG PET CT: discrete increase in glycolytic metabolism in the ILL (SUVmax = 5.7 / 5.1×3.7 cm), left lobe hilum (SUVmax = 3.0), hepatic hypovascular lesions (SUVmax = 5.6 / 2.2 cm in segment IVA and SUVmax = 6.6 in segment V), spine (SUVmax = 5.2 in D5), sacrum (SUVmax = 3.2) and pelvis (SUVmax = 3.3 left iliac). ^68^Ga-DOTATOC PET CT: significant increase of somatostatin receptor expression in the ILL (SUVmax = 22.7/5.1×3.7 cm), left lobe hilum (SUVmax = 17.3), hepatic hypovascular lesions (SUVmax = 25.1 in segment IVA and SUVmax = 37.3 in segment V), and multiple bone lesions, predominantly sclerotic, in the spine (SUVmax = 45.0 in D5), left sacrum (SUVmax = 3.2) and left pelvis (SUVmax = 12.6).

On pre-operative evaluation, a routine chest X-ray was performed that showed a 4 cm central lesion in inferior left pulmonary lobe (ILL). CT scan demonstrated a 4.5 × 4.0 × 3.5 cm lesion in ILL. ^18^Fluor-fluorodeoxyglucose positron emission tomography (^18^F-FDG PET) CT scan and ^68^Gallium DOTA-Tyr3octreotide (^68^Ga-DOTATOC) PET CT revealed discrete increase in glycolytic metabolism and significant increase of somatostatin receptor expression in the ILL, left lobe hilum, hepatic hypovascular lesions, spine, sacrum and pelvis, consistent with a well differentiated metastatic NET ( Figure 2 ). A bronchoscopy showed a vegetative and hypervascularized lesion, obstructing the left bronchial segment. The bronchial lesion was biopsied and was compatible with atypical pulmonary carcinoid (5 mitosis/2mm^2^; Ki-67 of 4%. No necrosis was seen.). At baseline, chromogranin A level was 17,906 ng/mL (normal range 25-140), serotonin 806.8 mcg/L (30-200) and urinary 5-hydroxyindolacetic acid 19.3 mg/24h (2.0-9.0). Serum GHRH concentration was not available for measurement.

### Histopathologic analysis of pituitary tissue

**Figure 3:**
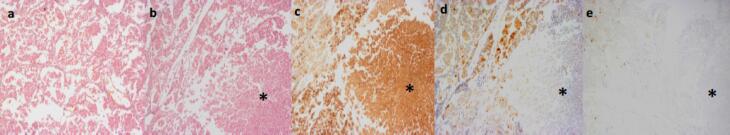
Histological sections showing enlarged lobules of the adenohypophysis (pituitary hyperplasia) in HE (a); pituitary hyperplasia and adenoma (*) in HE (b); GH positive in PA and pituitary hyperplasia (c); prolactin positive in pituitary hyperplasia (d); ACTH positive in pituitary hyperplasia (e).

Pathological examination included histological and immunohistochemical studies. For light microscopy, 3 μm sections of formalin-fixed and paraffin embedded tissue (FFPE) were stained with hematoxylin-eosin and reticulin. Avidin-biotin complex techniques were used to demonstrate the presence of pituitary hormones as described previously ( 9 ). Antisera were directed against adrenocorticotropic hormone (ACTH; dilution 1:6000, Cell Marque, Rocklin, Can, USA, cat. number 206A-76), GH (dilution 1:6000, Cell Marque, cat. number 208A-76), prolactin (PRL; dilution 1:7000, Cell Marque, cat. number 210A-16) and Ki-67 (clone MIB-1; dilution 1:5000, Cell Marque, cat. number 275R-16). Histopathological analysis identified a GH positive adenoma adjacent to pituitary hyperplasia characterized by enlarged lobular structure, in which GH cells predominated, while occasional PRL and ACTH immunostaining were also observed, confirming the non-neoplastic nature of this area ( Figure 3 ), despite tissue necrosis due to apoplexy. PRL and ACTH were negative in adenoma ( Figure 3 ). Ki-67 labeling index was 3.1%. Due to extensive tissue necrosis, other immunostains were not performed (LH, FSH, CAM5.2).

### Pulmonary tissue analysis

**Figure 4:**
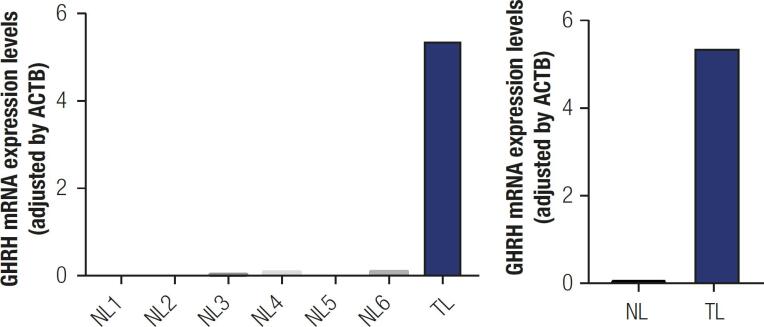
*GHRH* mRNA expression levels (adjusted by *ACTB* ).

RNA from the FFPE tissue was extracted from the pulmonary specimen, at a concentration of 13.6 ng/uL. Reverse transcription (RT) and quantitative polymerase chain reaction (qPCR) were performed as previously reported ( 10 ), showing very high expression levels of *GHRH* mRNA in the pulmonary NET (5053 mRNA copies) compared to six normal pulmonary tissues ( Figure 4 ). Three reference genes [ *beta-actin* ( *ACTB* ), *hypoxanthine guanine phosphoribosyl transferase* ( *HPRT* ) and *glyceraldehyde 3-phosphate dehydrogenase* ( *GAPDH* )] were also measured being *ACTB* the most stable control gene; therefore, *ACTB* was used to control for variations in the amount of RNA used in the RT reaction and to adjust the relative expression of each of the transcripts analyzed. *GH* was not expressed in the sample. The specific primers used in qPCR are detailed in Sup. Table 1 . At protein level, anti-GHRH (dilution 1:750, Abcam, cat. number ab187512) was also positive.

*ACTB:* beta-actin; NL1-6: normal lung tissue; TL: tumoral lung tissue.

### Medication

Levothyroxine, prednisone and testosterone were prescribed for hypopituitarism. Pegvisomant 70 mg/week and octreotide LAR 20 mg/month were started after ectopic acromegaly secondary to GHRH-producing metastatic tumor was diagnosed, while waiting for peptide receptor radionuclide therapy (PRRT). Octreotide LAR was titrated to 30mg and was given after ^177^Lu dose every 6 weeks. After four cycles of PRRT, ^18^F-FDG PET scan revealed complete metabolic resolution of the lesions and reduction of the primary pulmonary NET in about 22% in axial view. Pegvisomant was titrated to maintain IGF-I levels in normal range. Nowadays the patient is on pegvisomant 40 mg/week and octreotide LAR 30 mg/month. After one year of therapy, normalization of serum IGF-I to 197 ng/mL (99-289) and urine 5-hydroyindoleacetic acid to 8.9 mg/24h (2.0-9.0) were observed. Serum chromogranin A and serotonin levels decreased significantly but did not reach normal values [470 ng/mL (25-140) and 257.7 mcg/L (30-200), respectively]. Brain natriuretic peptide levels and echocardiography are normal.

### Genetic sequencing

Multiple endocrine neoplasia type 1 (MEN1) and 4 (MEN4) were investigated. Peripheral blood samples were collected with EDTA as anticoagulant and DNA was extracted from leukocytes. Somatic mutations were not analyzed due to paucity of tumor samples. Gentra PureGene Blood Kit (Qiagen, Minnneapolis, MN, USA) was used to obtain genomic DNA. The eluted was resuspended in 100 μL of DNA Hydration Solution following the manufacturer's instructions. After extraction, PCR reactions were performed on Applied Biosystem ProFlex™ PCR System (Thermofisher, Foster City, CA, USA). Amplification and sequencing were performed with PCR/Sanger Sequencing Primer pairs (ThermoFisher Scientific™, Boston, MA). Products were sequenced in both directions on ABI 3130xl Genetic Analyzer (Applied Biosystems) and electropherogram-derived sequences were aligned by using Benchiling and BioEdit software ( Sup. Table 2 ). Germline *MEN1 and cyclin dependent kinase inhibitor 1B (CDKN1B)* sequencing were negative for pathogenic mutations.

## DISCUSSION

The patient was initially diagnosed with pituitary adenoma (PA) and afterwards with pulmonary mass which turned out to be a GHRH-secreting pulmonary NET associated with somatotropinoma and pituitary hyperplasia. Because of extensive metastasis, primary pulmonary tumor was not resected and pegvisomant and octreotide LAR were started early, followed by PRRT. Compared to literature, our case is the only case in which PA and pituitary hyperplasia were observed in pathology, and pituitary hyperplasia was seen on MRI scans before pituitary adenoma formation.

To our knowledge, only 47 cases have been described of GHRH-secreting pulmonary NET causing acromegaly (48 including our case – Sup. Table 3 ). Literature review using PubMed and Google Scholar databases was performed with the following keywords: “GHRH-secreting tumor”, “bronchial carcinoid”, “pulmonary neuroendocrine tumor” and “ectopic acromegaly”. From literature review ( Sup. Table 3 ), GHRH-secreting pulmonary NET was more frequent in females representing 70.8 % of cases (34/48), which was not our case. Median age of the patients was 42.5 years (range: 19-77 years), older than our patient.

GHRH-secreting pulmonary NET were usually large tumors in most cases with a median diameter of 4.5 cm (1.2-9) on chest CT, like our case, and were more common on the right side [19 of 28 cases (67.8%) in which side was described], which was not our case. NETs were diagnosed by histology with the presence of positive immunostain for chromogranin and GHRH in the pulmonary specimen as found in our case, or suspected with positive functional imaging with increased serum GHRH. All patients except for three were submitted to pulmonary resection, even in the presence of metastatic lesions ( 11 , 12 ). Data on subtype of NET was available in 18 patients: 10 (55.5%) had typical pulmonary carcinoids and eight (44.5%) had atypical tumors.

In line with our case, 14 (33.3%) patients already had distant metastases at diagnosis. Most common sites were liver and/or bone present in 12 (75%) of these metastatic cases, similar to our case. Other sites included lymph nodes, breast, ovaries, heart, thyroid gland and even one case of metastasis to pituitary gland ( 13 ).

As NET can secrete bioactive amines such as serotonin, patients can present with carcinoid syndrome. This syndrome was uncommon in pulmonary NET (8% of patients) because of lower production of serotonin compared to other NET, but can be more frequently encountered in patients with liver metastases (15% of patients), as was our case ( 14 ). A delay of several years frequently occurred before a diagnosis of carcinoid tumor was made ( 15 ).

Pulmonary NET can also secrete GHRH causing acromegaly. An association may exist between ectopic acromegaly and genetic syndromes. Pulmonary NET has been reported in the context of MEN1 and MEN4 ( 16 ). Approximately 200 somatic *MEN1* mutations have been described in several different endocrine tumors, with 26 cases being of pulmonary NET ( 17 - 20 ). Although acromegaly can be seen in MEN1, in these pulmonary NET series it was not present. With respect to MEN4, one case of germline *CDKN1B* mutation who had pulmonary NET and pituitary adenoma has been described, but it was not of somatotroph origin ( 21 ).

Acromegaly was usually diagnosed before pulmonary NET as described in 33 patients of 48 cases (68.7%). Patients had signs and symptoms of acromegaly (prognathism, acral enlargement, hyperhidrosis) for median period of 8.5 years ( 2 - 26 ) prior to diagnosis. In these cases, patients had normal sellar imaging or findings suggestive of pituitary hyperplasia, so ectopic secretion was investigated. Interestingly, 12 patients already had a history of pulmonary mass or resected pulmonary tumor. Only five patients (10.4%) had local symptoms (pneumonitis or hemoptysis) and only four patients (8.3%) carcinoid syndrome. In five cases (10.4%), incidentally discovered pulmonary mass was seen on routine chest X-ray prior to surgery, as in our case.

In our case, pituitary hyperplasia was seen before adenoma formation, which has not been described previously. In the other cases, pituitary hyperplasia was seen during acromegaly investigation. It was present in 50% of patients who were submitted to brain or sellar imaging (CT or MRI). Eight (20%) cases were suspected of having pituitary tumor, but only five cases confirmed pituitary adenoma at histopathology examination (four somatotropinoma and one *null cell* adenoma) ( 5 , 13 , 22 , 23 ). Another patient might also have had an acidophilic cell adenoma or hyperplasia (indistinguishable) ( 24 ). In other five cases, patients were operated for a presumed somatotropinoma but histological examination only revealed pituitary hyperplasia (intact reticulin fiber network), suggesting that differentiation between pituitary hyperplasia and adenoma based on imaging was very difficult.

Pituitary adenoma and hyperplasia can coexist as we have seen in our case. In some reports, adenomatous transformation from pituitary hyperplasia have been described ( 5 ). In the five cases with pituitary adenoma, patients presented with acromegaly at earlier age [23 years old (19-44)] and was more common in men (3/5 cases), as seen in our case. Pulmonary NET was diagnosed incidentally in two cases including our own, or during investigation of metastatic tumor or of uncontrolled acromegaly ( 13 , 22 , 23 ). Apart from our case whose initial symptom was pituitary apoplexy, in the other cases, pulmonary NETs were present for at least 7.5 years ( 2 - 9 ) before a pituitary adenoma could be seen, which makes our case even more intriguing. Pituitary hyperplasia was observed on MRI before adenoma formation, which occurred relatively fast (for at least 20 months). We also presume our patient developed pituitary tumor rapidly because he presented with pituitary apoplexy. Rapid growth can precipitate apoplexy because tumoral blood vessels have inherent fragility contributing to hemorrhagic tendency ( 25 ). Additionally, another unique finding in our case was that of pituitary hyperplasia adjacent to adenomatous tissue on histopathology, rather than a mixture of hyperplasia and adenoma formation or a transition zone as seen in literature.

Biermasz and cols. ( 5 ) described a 27-year-old patient that had pituitary macroadenoma concomitant with GHRH-secreting pulmonary NET and had histopathological characteristics of a mixture of pituitary hyperplasia and adenoma formation in pituitary specimen. Also, Nasr and cols. ( 13 ) reported a case in which metastatic GHRH pulmonary NET to the pituitary was present adjacent to somatotroph hyperplasia and focal neoplastic transformation of sparsely granulated somatotroph phenotype suggesting a relation between GHRH hypersecretion and somatotroph cell disarrangement.

Similarly, in gangliocytoma (hypothalamic GHRH-producing tumor), somatotropinomas could be concomitantly seen ( 26 , 27 ). Moreover, in GHRH transgenic mice, histological findings resembled that of humans case reports in which diffuse pituitary hyperplasia and somatotropinoma can be found ( 28 , 29 ). Billestrup and cols. ( 30 ) reported that in anterior pituitary rat cells marked for GH, GHRH exposure lead to higher mitogenic index and GH secretion in these cells. These results were also seen when cells were treated with forskolin, which stimulates cyclic adenosine monophosphate (cAMP) production, mimicking GHRH receptor (GHRH-R) activation by its second messenger. Kineman and cols. ( 31 ) also reported in their study in mice that GHRH stimulation causes hypertrophic and hyperplastic effect in somatotrophs, also having found characteristic features of pituitary adenoma. Nevertheless, they believe the onset of pituitary adenomas was not preceded by a progressive increase in pituitary size, however these authors presume that when protective mechanisms are lost due to hyperactivation of GHRH-R in selected cells, adenoma formation occurs ( 31 ). Luque and cols. ( 32 ) showed that constitutive activation of GHRH-R in transgenic mice was associated with pituitary hyperplasia and adenoma formation late in life. These studies suggested that excess circulating GHRH could lead to pituitary hyperplasia and adenoma formation in animal pituitary.

In humans, a causative association between GHRH hyperstimulation and pituitary somatotroph adenoma formation has not been demonstrated. Theoretically, there may be neoplastic transformation preceded by pituitary hyperplasia. Ectopic GHRH excess usually results in pituitary hyperplasia alone ( Sup. Table 3 ) that has been described to be reversible ( 33 ). A hypothesis proposed is that the pituitary gland responds to trophic hormone secretion by undergoing reversible plastic changes in cell proliferation ( 34 ). Increased cell proliferation would lead to reversible hyperplasia or to pituitary adenoma formation depending on additional secondary genetic events. These events would be required for adenoma formation since not all cases develop pituitary adenoma ( 32 , 34 , 35 ).

Interestingly, other cases of somatotropinomas can also be associated with pituitary hyperplasia, as Villa and cols. ( 36 ) reported that tumors of non-identical twin sisters with *AIP* mutation had transition zones from hyperplasia to adenoma. Our case, however, did not show a transition zone from hyperplasia to adenoma in the pathological analysis, rather it showed pituitary adenoma adjacent to pituitary hyperplasia.

Regarding treatment, for acromegaly, pegvisomant is the drug with the highest efficacy and therefore was prescribed from the beginning for optimal and rapid IGF-I lowering ( 37 , 38 ). For pulmonary NET, surgical resection can cure the disease, however, in advanced metastatic cases, other options should be considered. Optimal management in the setting of advanced unresectable disease is not well established. Somatostatin receptor ligands (SRL) have been used in the treatment of ectopic acromegaly as they interrupt the pathophysiological process generated by ectopic GHRH secretion through suppression of tumoral GHRH and inhibition of GH secretion from the anterior pituitary gland ( 39 ). However, despite the favorable clinical improvement documented for some acromegaly patients, the response of GHRH-secreting tumor and its metastases is less predictable. For disseminated disease, such as our case, systemic therapy with radiolabeled somatostatin analogue therapy (also known as PRRT) can be appropriate. Lutetium has been studied mainly in gastroenteropancreatic NET in the NETTER-1 trial and in some series of pulmonary NET ( 40 , 41 ). Nevertheless, in a cost-effective, more economic approach, high doses of SRL ( 42 ) can be considered as monotherapy initially for NET with pegvisomant being prescribed after failure of SRL and PRRT.

In conclusion, very few cases of acromegaly secondary to GHRH-secreting pulmonary NET have been described, and even fewer of associated somatotropinoma. Despite its rarity, pituitary adenoma may coexist with pituitary hyperplasia in acromegaly due to ectopic origin. In the setting of acromegaly, ectopic GHRH or GH secretion must be thought of when pituitary scan shows pituitary hyperplasia or does not reveal a lesion suspicious of adenoma. Although generalizations cannot be made by case reports, we speculate, in our case, that the GHRH-producing pulmonary NET might have led to the development of pituitary hyperplasia and posteriorly to somatotropinoma formation that was manifested as PA.

## SUPPLEMENTARY

**Supplementary Table 1:** Primers used in qPCR

Gene	Sense	Antisense
*ACTB*	ACTCTTCCAGCCTTCCTTCCT	CAGTGATCTCCTTCTGCATCCT
*HPRT*	CTGAGGATTTGGAAAGGGTGT	TAATCCAGCAGGTCAGCAAAG
*GAPDH*	AATCCCATCACCATCTTCCA	AAATGAGCCCCAGCCTTC
*GH*	GACCTAGAGGAAGGCATCCAAA	AGCAGCCCGTAGTTCTTGAGTAG
*GHRH*	GTGATCCTCACCCTCAGCAA	TCGCTCTTGGTTGCTCTCTC

CTB: beta-actin; GAPDH: glyceraldehyde 3-phosphate dehydrogenase; GH: growth hormone; GHRH: growth hormone-releasing hormone; HPRT: hypoxanthine guanine phosphoribosyl transferase.

**Supplementary Table 2:** Genetic sequencing Codons of interest were amplified in 25 μL PCR reaction mixture. The amplified products were then visualized on 2% agarose gel stained with SYBR™ Safe DNA Gel Stain (Invitrogen by ThermoFisher Scientific). PCR products were purified from unincorporated nucleotides and primers using the ExoSAP-IT™ PCR Product Cleanup Reagent (Affymetrix Inc. by ThermoFisher Scientific). Direct sequencing of purified PCR products were performed in both directions on 3130XL Genetic Analyzer automatic sequencer (Applied Biosystems by ThermoFisher Scientific) and analyzed using the Benchiling software ( https://benchling.com/ ) and BioEdit ( http://www.mbio.ncsu.edu/BioEdit/bioedit.html ).

Exons	Sequence (5’->3’)	Product length	Exon	Gene	Cycling condition
CDKN1B_1Aexon_For	TGTAAAACGACGGCCAGTAATTTCTCCGAGGCCAGCCA	843	1	CDKN1B	94 °C for 5 min, 35 cycles of 94 °C for 45s, 59.5 °C for 45s, 72 °C for 50s, final extension of 72 °C for 7 min
CDKN1B_1Aexon_Rev	CAGGAAACAGCTATGACCCCCGGGTTAACTCTTCGTGG				
CDKN1B_1Bexon_For	TGTAAAACGACGGCCAGTTGTCAAACGTGCGAGTGTCT	543	1	CDKN1B	
CDKN1B_1Bexon_Rev	CAGGAAACAGCTATGACCAAGGTTAACACCCTCCAGCAG				
CDKN1B_2exon_For	TGTAAAACGACGGCCAGTCCCTTAAAAGCCACTGGGGAT	360	2	CDKN1B	
CDKN1B_2exon_Rev	CAGGAAACAGCTATGACCTGCCAGCAACCAGTAAGATCA				
CDKN1B_3Aexon_For	TGTAAAACGACGGCCAGTGTGCTCTTCCTTTAATTCTTCCG	779	3	CDKN1B	
CDKN1B_3Aexon_Rev	CAGGAAACAGCTATGACCCAAAACTCCCAAGCACCTCG				
CDKN1B_3Bexon_For	TGTAAAACGACGGCCAGTCTCCAGGTAGTTTGGGGCAA	596	3	CDKN1B	
CDKN1B_3Bexon_Rev	CAGGAAACAGCTATGACCATTGGCATCTTTTTCACACATTACA				
MEN1_01exon_For	TGTAAAACGACGGCCAGTTTAGCGGACCCTGGGAGGAG	227	2	MEN1	94 °C for 5 min, 35 cycles of 94 °C for 45s, 62 °C for 30s, 72 °C for 1 min, final extension of 72 °C for 7 min
MEN1_01exon_Rev	CAGGAAACAGCTATGACCTCCACGAAGCCCAGCACCAAG				
MEN1_02exon_For	TGTAAAACGACGGCCAGTCCTGTTTGCTGCCGAGCTGG	202	2	MEN1	
MEN1_02exon_Rev	CAGGAAACAGCTATGACCGGCGGCGATGATAGACAGGTC				
MEN1_03exon_For	TGTAAAACGACGGCCAGTCTGGCGGCCTCACCTACTTTC	152	2	MEN1	
MEN1_03exon_Rev	CAGGAAACAGCTATGACCGGAGACCTTCTTCACCAGCTCAC				
MEN1_04exon_For	TGTAAAACGACGGCCAGTGCCGTCGACCTGTCCCTCTATC	181	2	MEN1	
MEN1_04exon_Rev	CAGGAAACAGCTATGACCCATGGATAAGATTCCCACCTACTGG				
MEN1_05exon_For	TGTAAAACGACGGCCAGTGCACAGAGGACCCTCTTTCATTAC	197	3	MEN1	
MEN1_05exon_Rev	CAGGAAACAGCTATGACCCTTGCCGTGCCAGGTGAC				
MEN1_06exon_For	TGTAAAACGACGGCCAGTCTCGCCCTGTCTGAGGATCATG	190	3	MEN1	
MEN1_06exon_Rev	CAGGAAACAGCTATGACCTGGGTGGCTTGGGCTACTACAG				
MEN1_07exon_For	TGTAAAACGACGGCCAGTGGGCCATCATGAGACATAATG	192	4	MEN1	
MEN1_07exon_Rev	CAGGAAACAGCTATGACCCTGCCCCATTGGCTCAG				
MEN1_08exon_For	TGTAAAACGACGGCCAGTCCTGTTCCGTGGCTCATAACTC	160	5	MEN1	
MEN1_08exon_Rev	CAGGAAACAGCTATGACCCTAGGAAAGGATCATAATTCAGGC				
MEN1_09exon_For	TGTAAAACGACGGCCAGTGGGTGGCAGCCTGAATTATG	170	6	MEN1	
MEN1_09exon_Rev	CAGGAAACAGCTATGACCCTCAGCCACTGTTAGGGTCTCC				
MEN1_10exon_For	TGTAAAACGACGGCCAGTGGCTGCCTCCCTGAGGATC	251	7	MEN1	
MEN1_10exon_Rev	CAGGAAACAGCTATGACCCTGGACGAGGGTGGTTGG				
MEN1_11exon_For	TGTAAAACGACGGCCAGTGTGAGACCCCTTCAGACCCTAC	218	8	MEN1	
MEN1_11exon_Rev	CAGGAAACAGCTATGACCTGGGAGGCTGGACACAGG				
MEN1_12exon_For	TGTAAAACGACGGCCAGTGGGTGAGTAAGAGACTGATCTGTGC	246	9	MEN1	
MEN1_12exon_Rev	CAGGAAACAGCTATGACCTGTAGTGCCCAGACCTCTGTG				
MEN1_13exon_For	TGTAAAACGACGGCCAGTCGGCAACCTTGCTCTCACC	203	10	MEN1	
MEN1_13exon_Rev	CAGGAAACAGCTATGACCCCAGGCCCTTGTCCAGTG				
MEN1_14exon_For	TGTAAAACGACGGCCAGTGGGAGTCCAAGCCAGAGGAG	227	10	MEN1	
MEN1_14exon_Rev	CAGGAAACAGCTATGACCGCCCTTCATCTTCTCACTCTGG				
MEN1_15exon_For	TGTAAAACGACGGCCAGTTGCCAGCACCCGCAGCATC	253	10	MEN1	
MEN1_15exon_Rev	CAGGAAACAGCTATGACCCCCACAAGCGGTCCGAAGTCC				

CDKN1B: Cyclin Dependent Kinase Inhibitor 1B [p27(KIP1)]; MEN1: Multiple endocrine neoplasia type 1.

**Supplementary Table 3:** Literature review

Author, year	Age (in years), gender	Duration (symptoms and/or diagnosis)	Initial presenting diagnosis or symptoms	Sellar imaging	Pituitary pathology	Pulmonary NET investigation	Chest imaging	Type of pulmonary NET	Genetics or family history
Dabek, 1974	46, female	–	Acromegaly	X-Ray: sellar enlargement	–	During acromegaly investigation	Chest X-Ray: 6cm mass left lower lobe	Bronchial carcinoid	–
	30, female	–	Acromegaly	X-Ray: sellar enlargement	–	During acromegaly investigation	Chest X-ray: middle lobe mass	Bronchial carcinoid	–
Sonksen, 1976	60, female	–	Acromegaly	X-Ray: sellar enlargement	–	During acromegaly investigation + 19-year history of pulmonary symptoms (recurrent pneumonia)	Chest CT: right lower lobe mass	Bronchial carcinoid	–
	53, female	–	Acromegaly + pulmonary mass on routine X-ray	X-Ray: normal	–	During acromegaly investigation + pulmonary mass on routine X-ray	Chest X-Ray: right lower lobe mass	Bronchial carcinoid of 1.5 cm	–
Zafar, 1979	22, female	2 years	Acromegaly Recurrent pneumonitis	X-Ray: sellar enlargement	Acidophilic cell hyperplasia or adenoma	During persistent acromegaly + pulmonary symptoms	Bronchoscopy: right middle lobe mass	Bronchial carcinoid of 5×3×3 cm	–
Scheithauer, 1984	56, female	5 years 2 years	Acromegaly Cough, hemoptysis	CT: pituitary tumor	–	During acromegaly investigation + pulmonary symptoms	Chest X-Ray: obstructive lesion in right middle lobe	Metastatic bronchial carcinoid (lymph node) of 3.5 cm GHRH +	–
Hawkins, 1985	50, male	20 years 15 years	Lung mass Acromegaly	X-Ray: sellar enlargement with double floor CT: hyperdense image in the right half of the sellar space	–	During acromegaly investigation + history lung mass	Chest X-Ray: large opacity in the right middle zone	Bronchial carcinoid of 6×5×4 cm	–
Vance, 1985	48, male	20 years	Acromegaly	CT: normal	–	During acromegaly investigation + 19-year history of resected bronchial carcinoid	–	Metastatic lung carcinoid (liver, bone) GHRH +	–
Barkan, 1986	19, male	2 years	Acromegaly	CT: pituitary hyperplasia	Null cell microadenoma, somatotroph hyperplasia	During acromegaly investigation + history of metastatic lung tumor	–	Metastatic lung carcinoid (liver, bone) GHRH +	–
Boizel, 1987	55, female	24 years 22 years	Repeated hemoptysis Acromegaly	X-Ray: progressive mild sellar enlargement during 22 years CT: normal pituitary	–	Pulmonary symptoms + history of right postero-basal thoracic tumor	–	Bronchial carcinoid GHRH + 6cm right lower lobe	–
Carroll, 1987	29, female	8 years	Acromegaly	X-Ray: Increased sellar volume CT: normal	–	During acromegaly investigation	Chest X-ray: 3cm mass overlying the right heart border Present 6 years before	Bronchial carcinoid GHRH + 4cm right common lower lobe	Identical twin unaffected
Garcia-Luna, 1987	54, female	2 days 5 years 9 years	Pleurisy Hemoptysis Acromegaly	X-Ray: enlargement of pituitary fossa CT: “double floor”	–	Pulmonary symptoms	Chest X-ray: pleural effusion, mediastinal deviation Bronchoscopy: bleeding tumor distal bronchus intermedius	Metastatic bronchial carcinoid (lymph nodes) GHRH + 1.3cm Bronchus intermedius	–
Melmed, 1988	59, female	5 years	Acromegaly Acromegaly recurrence 2y after hypophysectomy	- CT: normal	Somatotroph hyperplasia	27-year history of resected bronchial carcinoid	Skeletal X-ray: multiple bone lesions	Metastatic bone tumor GHRH +	–
Moller, 1989	39, female	6 years	Acromegaly	CT: pituitary hyperplasia	–	13-year history left bronchial carcinoid resected	–	Metastatic bronchial carcinoid (breast, liver, pulmonary) GHRH+	–
Harris, 1990	19, female	- After 3 years After 4 years	Hemoptysis Carcinoid syndrome (diarrhea, flushing) Acromegaly (3 years after lobectomy)	X-ray: sellar enlargment	–	Pulmonary symptoms	Lower left lung lobe consolidation and right supraclavicular lymphadenopathy	Metastatic bronchial carcinoid (breast, lymph nodes, liver, bone, ovaries, cardiac, thyroid) GHRH +	–
Popovic, 1990	33, female	–	Acromegaly	–	–	–	–	Lung carcinoid GHRH +	–
Glikson, 1991	66, male	–	Acromegaly	CT: Normal	–	20-year history of right lung tumor	Chest CT: large well circumscribed tumor in the right lower lobe	Bronchial carcinoid GHRH + 7.5 × 6 × 6 cm	–
Ezzat, 1994	28, female	Long-standing	Acromegaly	MRI: pituitary hyperplasia	Somatotroph hyperplasia	10-year history of resected bronchial carcinoid (left)	–	Metastatic endobronchial carcinoid (breast, chest wall, ovaries) GHRH + Chromogranin +	–
Lefebvre, 1995	21, female	2 years	Acromegaly	MRI: pituitary hyperplasia	–	3-year history of resected bronchial carcinoid (right)	–	Metastatic bronchial carcinoid (lymph node, lung) GHRH +	–
Platts, 1997	53, female	13 years 4 years -	Pulmonary mass found during pneumonia Hemoptysis Acromegaly	CT: normal	–	Pulmonary symptoms	Chest X-ray: 9cm stable right hilar mass (over 13y period) Positive 111In-labeled-octreotide scintigraphy	Bronchial carcinoid GHRH +	–
Drange, 1998	42, male	17 years	Acromegaly	MRI: 1.0 × 1.8 × 2.0 cm pituitary mass	–	28-year history of resected bronchial carcinoid	Positive 111In-labeled-octreotide scintigraphy for metastasis *no imaging on initial diagnosis	Metastatic bronchial carcinoid (bone, liver)	–
Othman, 2001	39, male	10 years	Acromegaly	MRI: pituitary hyperplasia	Somatotroph hyperplasia	During persistent acromegaly	Positive 111In-labeled-octreotide scintigraphy: globular mass in right lower lobe of the lung	Bronchial carcinoid GHRH + 10 cm	
Bhansali, 2002	38, female	3 days few months	Pleuritic chest pain Acromegaly	MRI: pituitary hyperplasia	–	Pulmonary symptoms	Chest CT: right hilar mass	Bronchial carcinoid GHRH + Right upper lobe 3 × 3 cm	–
Bolanowski, 2002	61, female	10 years	Acromegaly, carcinoid syndrome (weight loss, diarrhea)	MRI: pituitary hyperplasia	–	During investigation of acromegaly and carcinoid syndrome	Chest X-ray: round 10 cm lesion based on diaphragm in the back region of right lung Chest CT: 9 × 7cm lesion in the postero-medial part of right lung	Typical bronchial carcinoid Chromogranin + GHRH+ (Serum)	–
Osella, 2003	47, female	–	Acromegaly	MRI: empty sella	–	During acromegaly investigation	Chest CT: 3.5 cm right lower lung mass Positive 111In-labeled-octreotide scintigraphy	Atypical bronchial carcinoid GHRH +	Positive family history for bronchial carcinoid
Reuters, 2003	61, female	–	Acromegaly	MRI: pituitary hyperplasia	–	During pulmonary mass investigation	Chest CT and MRI: 8 cm mass	Typical bronchial carcinoid GHRH +	–
Athanassiadi, 2004	37, male	8 years	Acromegaly	–	1.3 × 1.3 cm GH + PRL adenoma	During persistent acromegaly	Chest X-Ray: large tumor in left lung Chest CT: large circumscribed tumor in the left upper lobe	Bronchial carcinoid Gastrin + Somatostatin + 6.0×5.5×4.5 cm	–
Zatelli, 2005	29, female	10 years	Acromegaly	MRI: pituitary hyperplasia	–	During acromegaly investigation	Chest X-ray/MRI: 3.3 cm basal portion of the left superior pulmonary lobe Positive 111In-labeled-octreotide scintigraphy	Atypical bronchial carcinoid	–
Nasr, 2006	44, female	2 week 7 years later -	Scalp lesion Bitemporal hemianopia Intermitent watery stools once or twice a month	MRI: 2.6 cm pituitary tumor	Metastatic GHRH+ tumor and somatotroph hyperplasia with focal neoplastic transformation	During metastatic lesion investigation (scalp biopsy: neuroendocrine cells)	Chest X-Ray: lobulated 3 cm right hilar soft tissue mass Chest CT: 2.5cm right infrahilar mass	Metastatic atypical pulmonary carcinoid (bone, chest, abdomen, pituitary) GHRH +	–
Biermasz, 2007	27, female	5 years	Acromegaly	MRI: pituitary hyperplasia	–	During investigation of acromegaly	Chest X-ray: large tumor in the right lower lobe of the lung. Positive 111In-labeled-octreotide scintigraphy	Metastatic lung carcinoid (liver) Chromogranin + Synaptophysin + 5 cm	–
	27, male	9 years	Acromegaly	MRI: pituitary tumor	Mixture of hyperplasia and GH+PRL adenoma	Incidental during pre-operative pituitary surgery	Chest X-ray: large parahilar lesion in left lung Positive 111 In-labelled-octreotide scanning	Atypical bronchial carcinoid Synaptophysin + 8×7×7cm	–
Fainstein Day, 2007	36, male	3 years	Acromegaly	MRI: pituitary hyperplasia	–	19-year history of resected bronchial carcinoid	Chest CT: 1.2cm right parahilar region	Metastatic atypical bronchial carcinoid (lung, liver, bone, brain) GHRH +	–
De Jager, 2007	74, female	- 15 years	Anemia from colon adenocarcinoma Acromegaly	MRI: pituitary hyperplasia	–	15-year history of stable solid lesion of the right lung	Somatostatin receptor scintigraphy 111In pentetreotide	Biopsy: atypical cells compatible with carcinoid tumor	–
Van Hoek, 2009	56, female	3 years >5 years	Acromegaly Cough, hemoptysis	MRI: normal	–	During acromegaly investigation + pulmonary symptoms	Chest X-Ray and CT: pulmonary paramediastinal 4cm mass in the right-upper lobe Positive octreoscan: right hemitorax	Typical bronchial carcinoid GHRH +	–
Verrua, 2010	55, female	–	Acromegaly	MRI: pituitary hyperplasia, uncertain focal lesion	–	Incidental during pre-operative pituitary surgery	Chest X-Ray: large thickening in left lung Chest CT: 6.0×8.4×5.0 cm mass in contact with pulmonary hilum. Positive octreoscan (111In pentatreotide)	Typical bronchial carcinoid GHRH +	–
Gudbjartsson, 2011	42, female	–	Acromegaly	MRI: pituitary hyperplasia	–	Incidental during pre-operative pituitary surgery	Chest X-Ray: 7cm right-sided lung tumor	Typical bronchial carcinoid GHRH + Between intermediate and lower right bronchus	–
Butler, 2012	54, male	20 years -	Acromegaly Hemoptysis	MRI: pituitary hyperplasia	–	History of lobectomy	Chest CT: 4.4 cm mass adjacent left hilum Positive octreoscan	Bronchial carcinoid Chromogranin + GHRH + 3.3 cm	–
Garby, 2012	7 patients with bronchial carcinoid 30 female 30 female 27 female 28 male 42 female 53 female 53 male 77 female	–	Acromegaly	MRI: pituitary hyperplasia in 4/7 cases and normal in 3/7 cases	1 operated case: somatotroph hyperplasia	During investigation of ectopic GHRH secretion	Chest CT 1. 45 mm 2. 40 mm 3. 12 mm 4. 60 mm 5. 15 mm 6. 80 mm 7. 70 mm	typical carcinoid typical carcinoid (serotonin+ calcitonin +) metastatic typical carcinoid (liver, bone) typical carcinoid typical carcinoid metastatic atypical carcinoid (liver, bone) -	patient tested for *MEN1* germline mutation: negative
Rojo Alvaro, 2013	38, female	8 years 2 years	Acromegaly Carcinoid syndrome (facial flushing, tachycardia)	MRI: normal	–	During acromegaly investigation	Chest X-Ray: right hilum mass Chest CT: 7 × 4 × 3.5 cm right hilum mass Positive octreoscan	Bronchial carcinoid Chromogranin + 5 × 3.5 cm	–
Kyriakakis, 2017	52, male	26 years 22 years 17 years	Acromegaly symptoms Acromegaly confirmed, abdominal pain Spinal surgery	CT: pituitary tumor	Pituitary adenoma (initially) Strong positivity for GH and PRL Scattered positivity for LH, FSH, TSH, ACTH Pituitary hyperplasia (after re-examination of pituitary specimens)	Incidental during pre-operative spinal surgery	Chest X-ray: right lower lobe shadow Chest CT: 8 cm tumor in the posterior segment of the right lower lobe	Bronchial carcinoid Chromogranin + GHRH +	–
Stelmachowska-Banas, 2019	43, female	2 years	Acromegaly symptoms	MRI: pituitary hyperplasia	–	During acromegaly investigation	Chest X-ray: 5.6 × 4.0 cm tumor in right anterior mediastinum Chest CT: 5 cm tumor Positive octreoscan	Typical bronchial carcinoid Chromogranin + GHRH +	–
Current case	22, male	Short-lasting 1 year	Pituitary apoplexy Abdominal cramps	Initial MRI: Pituitary hyperplasia 2^nd^MRI: pituitary tumor	Pituitary hyperplasia and somatotropinoma	Incidental during pre-operative pituitary surgery	Chest X-ray: central lesion in inferior left pulmonary lobe (ILL) Chest CT: 4.5 × 4.0 × 3.5 cm lesion in ILL Positive Ga-PET CT	Metastatic atypical carcinoid (liver, bone) Chromogranin + GHRH +	Negative *MEN1* and *CDKN1B* germline mutations

CDKN1B: Cyclin Dependent Kinase Inhibitor 1B [p27(KIP1)]; CT: computed tomography; GH: growth hormone; GHRH: growth hormone-releasing hormone MEN1: Multiple endocrine neoplasia type 1; MRI: magnetic resonance imaging; PRL: prolactin.
